# Sex Differences and Aging

**DOI:** 10.1093/geroni/igaf122.1531

**Published:** 2025-12-31

**Authors:** Brittany Lasseigne

## Abstract

Sex differences are critical for deciphering normal biological function, including the aging process. However, the biological influence of sex, including metabolic, genomic, epigenetic, immunological, hormonal, and other differences between males and females, has been under-addressed in biomedical research. This gap has prompted key policies, notably the National Institutes of Health’s Sex as a Biological Variable (SABV) and the Sex and Gender Equity in Research (SAGER) guidelines, designed to motivate researchers to consider sex differences in their studies. This session focuses on the impact of sex across the lifespan. Our presentations and discussion will center on identifying and characterizing sex-specific molecular signatures of aging, their role in developmental programming, and their influence on tissue-specific aging. We will also examine how biological sex modifies the effects of lifestyle interventions like exercise on the aging trajectory.

